# Subjective Happiness and Compassion Are Enough to Increase Teachers’ Work Engagement?

**DOI:** 10.3389/fpsyg.2019.02268

**Published:** 2019-10-17

**Authors:** Simona De Stasio, Caterina Fiorilli, Paula Benevene, Francesca Boldrini, Benedetta Ragni, Alessandro Pepe, Juan José Maldonado Briegas

**Affiliations:** ^1^Department of Human Sciences, Libera Università Maria SS. Assunta, Rome, Italy; ^2^Department of Human Studies University of Milano-Bicocca, Milan, Italy; ^3^Department of Business Management and Sociology, University of Extremadura, Badajoz, Spain

**Keywords:** subjective happiness, compassion, work engagement, Italian early childhood teachers, proactive strategies

## Abstract

The present quantitative multi-trait cross-sectional study aims to gain a better understanding of the network of relationship between subjective happiness, compassion, levels of work engagement, and proactive strategies (self- and co-regulation) in a sample of teachers. Participants were 187 full-time in-service teachers (89% female; age *M* = 48.5; *SD* = 7.88) from Rome, Italy. We hypothesized that subjective happiness and compassion of early childhood teachers would be related with work engagement in such a way that subjective happiness would promote the engagement of teachers. In a similar fashion, we theorized that subjective happiness would be positively related to self- and co-regulation strategies and that proactive strategies would be in turn associated to work engagement. As expected, the results revealed that subjective happiness and compassion showed effects on work engagement and that this association among constructs was mediated by the role of proactive strategies (β = 0.22, *p* < 0.001; β = 0.37, *p* < 0.001, respectively). Proactive strategies also have a significant direct effect on work engagement (β = 0.56, *p* < 0.001). The study’s findings suggest the importance of investing in the quality of the working environment.

## Introduction

Recent studies suggest that the relational nature of the early childhood education work environment bears key implications for teachers’ well-being. Teaching is conducted in a social setting that involves not only direct interaction with children and their parents (Kremenitzer and Miller, 2008) but also cooperation with colleagues, head teachers, and other workgroup members (Leiter et al., 2012). According to Johnson et al. (2012), teachers see the school work environment as comprising three main components: working relationships with colleagues, the school principal’s leadership, and school culture, all of which can contribute to teacher work satisfaction and retention rates. Teachers’ everyday social interactions in the workplace are characterized by major emotional involvement, while positive relational bonds can represent a crucial protective factor for their job satisfaction and well-being (Saarni, 1999; Albanese et al., 2014; Fiorilli et al., 2015; Benevene et al., 2018a). The literature recognizes that individual dispositional variables, such as perceived subjective well-being and compassion toward others, influence the quality of teachers’ working experience (Hargreaves, 2000; Morgeson and Humphrey, 2006; Warr, 2007; Boehm and Lyubomirsky, 2008; Lilius et al., 2008; Underwood, 2009; Benevene et al., 2018b). A positive atmosphere at school fosters job satisfaction and work engagement (Hoy and Spero, 2005), which, in turn, enhance job performance (Xanthopoulou et al., 2009). A number of studies found that teachers benefit from being compassionate, suggesting that affect plays a key role in work engagement (Brief and Weiss, 2002; Barsade et al., 2003; Hareli and Rafaeli, 2008; Robinson et al., 2013). Furthermore, Nislin et al. (2016) reported that good quality teamwork among teachers helps them to select effective strategies for coping with stressful work conditions. It is therefore of interest to investigate whether dispositional variables, such as subjective happiness and compassion, together with socio-contextual factors, such as self/co-regulated proactive strategies, predict the work engagement of early childhood (0–6 years) teachers.

Fredrickson’s (2001) “*Broaden-and-build theory of positive emotion*” suggests that frequent positive affect triggered by subjective happiness and compassion at work will influence teachers’ work outcomes. According to this theory, experiencing positive emotions at work contributes to broadening workers’ individual mindsets, enabling them to build up their personal resources in terms of enhanced sensitivity and positive attitudes toward their workplace (Fredrickson, 2013). In educational settings, experiencing positive affect can lead early childhood teachers to form positive emotional associations with their workplace, progressively helping them to view it more positively, and fostering their emotional vigor and organizational commitment (Fredrickson, 2001; Fredrickson et al., 2003).

## Dispositional Variables and Work Engagement

Personal experience in the workplace can be influenced by multiple individual factors: among these, subjective happiness and dispositional positive affect toward others have recently been shown to wield a particularly strong influence (Burger and Caldwell, 2000; Morgeson and Humphrey, 2006; Warr, 2007; Boehm and Lyubomirsky, 2008; Lilius et al., 2008).

Happiness may be viewed as a dispositional measure of subjective well-being; it can help to explain why some individuals report greater self-perceived well-being as a function of life changes, while others report the same amount of well-being regardless of life events (Lyubomirsky and Lepper, 1999). Some authors use the term “subjective well-being” in preference to “happiness”, on the grounds that the former has been more precisely defined in the scientific literature (Diener et al., 2017). In 1999, Lyubomirsky and Lepper introduced the concept of “subjective well-being” as a dispositional factor capable of explaining individual differences in coding, interpreting, and responding emotionally to life events (Lyubomirsky, 2001). A body of research suggests that perceived subjective well-being can influence the way people adapt to situations, events, and everyday life (Lucas, 2007; Luhmann et al., 2012).

As posited by the above-mentioned “Broaden-and-Build-Theory of positive emotions” (Fredrickson, 2001), subjective well-being contributes to predicting how people will manage and experience their work environment (Lyubomirsky et al., 2005). According to Fredrickson (2001), frequent positive affect leads people to broaden their cognitive and behavioral repertoire and thereby to reinforce their store of personal resources such as self-efficacy, resilience, and optimism. Studies have shown that high dispositional subjective well-being is associated with positive work outcomes, superior performance, success, and higher levels of perceived social support from colleagues (Staw and Barsade, 1993; Staw et al., 1994; Burger and Caldwell, 2000; Boehm and Lyubomirsky, 2008). Happier workers are more likely to engage in altruistic and cooperative behaviors, thus enhancing both the general atmosphere and all-around productivity in the workplace (Borman et al., 2001; Lee and Allen, 2002; Miles et al., 2002; Ilies et al., 2006; Boehm and Lyubomirsky, 2008). They also learn to manage workload and stress more effectively, and make better work-related decisions (Staw and Barsade, 1993; Iverson et al., 1998; Morgeson and Humphrey, 2006; Warr, 2007).

Although several studies have identified a relationship between subjective happiness and work engagement, this association has yet to be investigated in-depth in relation to school and educational settings (Bakker and Oerlemans, 2016). In one of the few studies on the topic, Kim and Shin (2016) noted that early education teachers’ happiness contributes to predicting their educational strategies, the quality of their interaction with the children, and their ability to foster positive social interaction in the classroom.

Among the dispositional variables that can affect workplace well-being, “compassion” is widely described in the literature as comprising three components: perceiving other people’s suffering; reacting to it in terms of empathizing with the other person’s discomfort; and offering a behavioral response with the aim of alleviating the other’s suffering (Davis, 1983; Clark, 1997; Kanov et al., 2004; Dutton et al., 2006; Miller, 2007). Lilius et al. (2008) reported that compassionate behaviors in the workplace can have long-lasting effects on how individuals experience their work; compassion may be expressed by providing colleagues with emotional support, or by facilitating them in organizing their work, for example, by allowing them more flexible working hours (*ibidem*).

Although compassionate behaviors are a characteristic feature in many occupational contexts (Eldor and Shoshani, 2016), only a limited number of studies have examined the relationship between compassion and work-related experience (O’Brien, 2006; Tehan and Robinson, 2009).

Early education settings are particularly dependent on compassionate conduct, given that teachers provide crucial care to infants and children (Kremenitzer, 2005; Sutton and Wheatley, 2003; Kremenitzer and Miller, 2008). Compassion in the education sector has mainly been viewed as an expression of a teacher’s attitude toward his/her pupils. However, this is an unduly restrictive definition: teachers also express compassion toward other adults such as their teaching colleagues, and thus can also be the recipients of affection and positive emotions. Compassion among teachers can enhance their feelings of emotional connection to their work and strengthen the association between organizational support and work commitment (Eldor and Shoshani, 2016). Some authors have investigated how compassion and proactive coping strategies are related to one another, as well as their effects on teacher adaptability and job satisfaction, and general classroom atmosphere (Mason et al., 2014; Mauno et al., 2016).

## The Role of Proactive Strategies in Early Childhood Teachers’ Work Environment

In the school setting, the term “coping strategies” refers to teachers’ cognitive and behavioral efforts to reduce, tolerate, or manage work-related stress (Lazarus, 1993; Sharplin et al., 2011; Parker et al., 2012).

“Self-regulated” coping strategies are generated by individual teachers, with a view to managing stressful work situations (Zimmerman, 2002; Boekaerts and Corno, 2005). When teachers manage stressful scenarios by drawing on the social resources made available to them by their community of colleagues, these strategies may be defined as “co-regulated” (Järvelä et al., 2010).

Considering the highly social nature of educational and school settings, it is reasonable to assume that teachers typically co-regulate their behavior in order to effectively cope with stressful conditions. Soini et al. (2010) found that, in schools, teachers who feel positively toward their work environment often use externally oriented problem-solving strategies and co-regulate their behavior with colleagues. Teachers who report high levels of support from colleagues are less likely to intend giving up teaching, as compared to those who report finding low levels of social support in the work setting (Pomaki et al., 2010). Proactive strategies help individuals to appropriately direct their behaviors, attain pre-set goals, and reduce their risk of burnout (Xanthopoulou et al., 2009; Devos et al., 2012).

When teachers experience job satisfaction and motivation, this generates a positive social atmosphere (Alhija and Fresko, 2010; Skaalvik and Skaalvik, 2011). According to Fredrickson (2001), positive affect fosters more creative and flexible use of available strategies. Teachers who are engaged with their work are significantly more inclined to deploy active and reflective approaches to problem-solving (Soini et al., 2010). The use of proactive strategies not only reduces the risk of burnout but also contributes to the development of a positive work environment and to increased job involvement. Their degree of efficacy appears to be closely related to the quality of teachers’ social interactions, suggesting that co-regulation plays a key role in prompting and sustaining proactive intervention.

## Work Engagement

Work engagement may be defined as “*a state of fulfilment characterized by vigor, dedication, and absorption*” (Schaufeli et al., 2002). The term “vigor” describes an energetic, effortful, and persistent approach to one’s work; “dedication” denotes a high level of involvement, accompanied by a sense of meaningfulness, enthusiasm, and inspiration; the term “absorption” indicates concentration and feeling engrossed in one’s work, such that the time spent working passes quickly (*ibidem*).

Work engagement is a predictor of work-related well-being, at both the individual and organizational levels (Bakker et al., 2008). In the school setting, work engagement strongly predicts job performance, reduced intention to quit teaching, positive teacher–student relationships, and student academic achievement (Bakker et al., 2006, 2008; Duckworth et al., 2009; Salanova et al., 2010).

Potential links between individual dispositional variables, such as subjective well-being and levels of work-engagement, have been explored in the literature (Field and Buitendach, 2011; Huynh et al., 2014; Rodríguez-Muñoz et al., 2014; Bakker and Oerlemans, 2016), but inadequately so in relation to educational and school settings (Pillay et al., 2005; Chan, 2009). Indeed, to date, subjective well-being in teachers has been viewed as an outcome of work engagement, rather than one of its potential antecedents (Tadić et al., 2013; Sirisunhirun and Dhirathiti, 2015).

Among individual dispositional resources, compassion at work has emerged as a strong predictor of work engagement (Mason et al., 2014; Mauno et al., 2016), being positively associated with teachers’ levels of vigor, dedication, and satisfaction. It is also negatively associated with burnout, reflecting its status as a key resource for coping with stress-related burden (Eldor and Shoshani, 2016). Receiving constructive feedback and professional recognition from colleagues and superiors significantly affects job performance (Klusmann et al., 2008; Xanthopoulou et al., 2009), promoting job engagement and satisfaction (Peeters and Rutte, 2005; Kokkinos, 2007; Stoeber and Rennert, 2008).

## Aims of the Study

The present quantitative multi-trait cross-sectional study aims to gain a better understanding of the network of relationship between subjective happiness, compassion, levels of work engagement and proactive strategies (self- and co-regulation) in a sample of early childhood teachers. More specifically, we were first interested in evaluating the degree of fit of an integrated structural model with empirical data and then to assess the cumulative network by decomposing total effects in direct and indirect effects among considered variables.

Based on previous research, we expected that subjective happiness and compassion of early childhood teachers would be related with work engagement in such a way that subjective happiness would promote the engagement of teachers. In a similar fashion, we theorized that subjective happiness would be positively related to self- and co-regulation strategies and that proactive strategies would be in turn associated to work engagement. Finally, and most germane for the present paper, the representation of the network of association in an integrated structural model supported the theoretical viewpoints (Borrelli et al., 2014) considering subjective happiness, work engagement as dynamically shaping the social endeavor in which teachers are involved during their daily profession.

## Materials and Methods

### Participants and Procedure

Our sample was composed of 187 full-time in-service teachers (89% female) from Rome, Italy. Ages ranged from 27 to 63 (*M* = 48.5; *SD* = 7.88) In terms of marital status, 56.5% were married, 21.6% were single, 18.4% were separated/divorced, and 1.6% were widowed; 65% of participants had children. Length of teaching experience ranged from 1 to 32 years (*M* = 17.23 years, *SD* = 14.23). The study population was a convenience sample and may not be taken as representative of the entire population of Italian teachers given that all participants were based in Central Italy. The authors organized plenary assemblies in kindergarten schools to inform the teachers about the aims of the research and the procedures for completion of the questionnaires. All participants signed informed consent forms and were assured anonymity and confidentiality. The research protocol was approved by the Ethics Committee of LUMSA University, Rome. The original versions of questionnaires were initially translated from English into Italian and then back-translated into English to check the alignment with the original versions.

### Measures

The Subjective Happiness Scale (Lyubomirsky and Lepper, 1999) is a four-item scale that assesses subjective happiness, using a seven-point Likert scale. The first two items ask people to rate how they are generally happy about their life (1 = not a very happy person, 7 = a very happy person) and how happy they are in comparison with their peers (1 = less happy, 7 = more happy) (e.g., “Compared with most of my peers, I consider myself less happy … more happy”).

The last two items ask respondents to what extent the characterization of a happy and of an unhappy person describe themselves (1 = *not at all*, 7 = *a great deal*). Higher scores on this measure indicate greater subjective happiness. It is utilized in the Italian version adapted by De Stasio et al. (2017). Prior studies have reported Cronbach’s alpha coefficients for the SHS from 0.79 to 0.94 (Lyubomirsky and Lepper, 1999).

The Santa Clara Brief Compassion Scale (Hwang et al., 2008) is a five-item scale; it assesses compassion and its link to pro-social behaviors. The scale includes items related to two facets of compassion: “emotionally connecting with other people’s suffering” (e.g., “When I hear about someone going through a difficult time, I feel a great deal of compassion for him or her”) and “acting to help them” (e.g., “I often have tender feelings toward people when they seem to be in need”). It is a shortened version of Sprecher and Fehr’s (2005) Compassionate Love Scale (the correlation between the two scales is *r* = 0.95) and it refers to non-intimate (i.e., strangers) rather than to close others. It is utilized in the original version translated in Italian through back-translation carried out by the authors. All items were rated on a seven-point Likert-type scale ranging from 1 (completely disagree) to 7 (completely agree), and higher scores are indicative of greater compassion. Cronbach’s alpha was 0.87.

The Utrecht Work Engagement Scale [UWES-17; Schaufeli et al., 2006, Italian version of UWES-17, validated by Balducci et al. (2014)] assesses work engagement. The scale is composed of 17 items, grouped into three subscales, namely, vigor (six items), dedication (five items), and absorption (six items) (e.g., “At my job, I feel strong and vigorous”). All items are scored on a seven-point frequency rating scale ranging from 0 (never) to 6 (always). Cronbach’s alpha was 0.96.

The Proactive Strategy scale (Salmela-Aro, 2009) consists of seven items, measuring two factors of proactive strategies: (a) self-regulation (4 items) and (b) co-regulation (3 items), meaning, respectively, the ability to identify and use resources for coping with stressors and the ability to seek and receive social support from colleagues (e.g., “I can set limits to my work assignments”; “I’m able to support the colleagues who feel strain in their work”). It is utilized in the original version translated in Italian through back-translation carried out by the authors. All items are rated on a seven-point Likert-type scale, ranging from 1 (completely disagree) to 7 (completely agree). Cronbach’s alpha was 0.67.

### Analytical Strategy

#### Preliminary Analysis

First, chi-square tests and correlational analysis were conducted to identify potentially confounding interrelationships among participants’ demographic characteristics (age and gender) and the measures under study. Second, the statistical distribution of the data was explored for each of the measures. None of the kurtosis and skewness values were in excess of the recommended cutoffs [−1, +1]. Mahalanobis’ distance (*p* < 0.001) was calculated for all scores in order to identify and skip any multivariate outliers. As a result, one extreme multivariate value was omitted from the analyses.

#### Structural Equation Modeling

The cumulative network of relationships among the variables of interest was analyzed via structural equation modeling (SEM), implemented using AMOS (Arbuckle and Wothke, 2006), version 21.0. This analytical strategy involves statistically testing a hypothesized set of direct and indirect paths among variables to evaluate the extent to which it fits the empirical data, yielding a measure known as goodness of fit. In the current study, we followed standard recommendations for the evaluation of a given SEM model (Bagozzi and Edwards, 1998; Hu and Bentler, 1999; Hair et al., 2010) by adopting two different types of fit index: absolute and relative. The absolute indexes selected were χ^2^ and normed-χ^2^ (NC) [a non-statistically significant χ^2^ value and NC values of under 2.0 indicate good fit (Hair et al., 2010)]. The relative indices adopted were the root mean square error of approximation (RMSEA), normed fit index (NFI), non-normed fit index (NNFI), comparative fit index (CFI), and standardized root mean square residual (SRMR). In this case, the thresholds set for good model fit were: RMSEA < 0.07 (Schermelleh-Engel et al., 2003), NFI > 0.95, NNFI > 0.95 (Marsh, 2004), and CFI > 0.95 (Hu and Bentler, 1999). Finally, in keeping with current literature on the use of SEM (e.g., MacKinnon et al., 2004), we estimated confidence limits using both Monte Carlo simulation and bootstrapping methods with a set of random samples (*k* = 500).

## Results

Table 1 reports correlations and descriptive statistics (means and standard deviations) for all the variables under study. Subjective happiness was statistically significant and slightly correlated with teachers’ self-regulation strategies (*r* = 0.15, *p* < 0.005), co-regulation strategies (*r* = 0.19, *p* < 0.001), and work engagement (*r* = 0.21, *p* < 0.001). In a similar fashion, the correlations between compassion and work engagement (*r* = 0.42, *p* < 0.001), teachers’ self- regulation (*r* = 0.28, *p* < 0.001), and co-regulation strategies (*r* = 0.31, *p* < 0.001) were positive and statistically significant. Both teachers’ self-regulation (*r* = 0.38, *p* < 0.001) and co-regulation (*r* = 0.47, *p* < 0.001) strategies were significantly correlated with work engagement.

**TABLE 1:** Bivariate correlations and descriptive statistics (means and standard deviations) for all the variables under study.

	**1**	**2**	**3**	**4**	**5**
1. Work engagement	1				
2. Subjective happiness	0.205^∗∗^	1			
3. Compassion	0.420^∗∗^	0.147	1		
4. Co-regulation strategies	0.468^∗∗^	0.194^∗∗^	0.310^∗∗^	1	
5. Self-regulation strategies	0.385^∗∗^	0.151^∗^	0.276^∗∗^	0.435^∗∗^	1
M	84.3	19.8	29.4	18.1	22.4
S.D.	13.1	3.8	4.1	2.2	3.8

*^∗∗^*p* < 0.01; ^∗^*p* < 0.05.*

**FIGURE 1:**
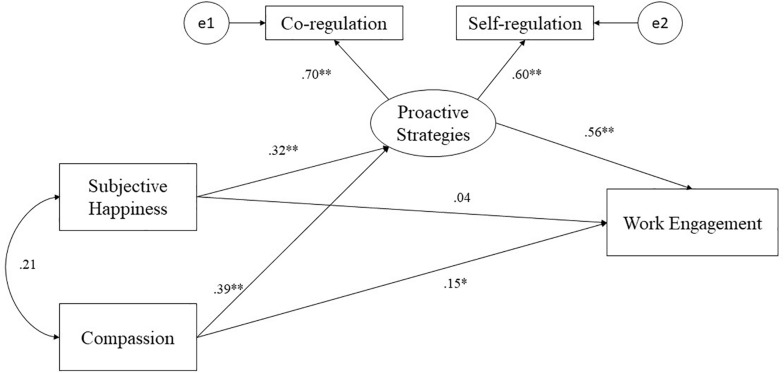
Results of the structural equation model. Standardized direct effect were reported. ^∗^*p* < 0.05, ^∗∗^*p* < 0.01.

The estimation of the conceptual model (see Figure 1) allowed us to test the hypothesis that the pattern of association between measures of subjective happiness, compassion, proactive strategies, and work engagement can be modeled within a single integrated model. SEM revealed that relative indexes of fit were generally robust [χ^2^ (33) = 0.279, *p* = 0.870; NC = 0.14], suggesting a good degree of fit between the conceptual model and the actual empirical data. Further information about the practical significance of the model was provided by analysis of absolute indexes (RMSEA = 0.003, *p*_close_ = 0.913, SRMR = 0.001, NFI = 0.998, CFI = 0.999), all of whose values fell within the recommended cutoff points. The analysis of total effects (standardized weights) and the subsequent decomposition in direct and indirect effects estimated by the structural equations model allowed us to explore more specific research hypotheses. The results suggested that subjective happiness wielded a positive total effect [β = 0.22, *p* = 0.005, 95th C.I. (0.607–1.18)] on work engagement, composed of a non-statistically significant direct effect [β = 0.04, *p* = 0.590, 95th C.I. (-0.209–0.448)] and of an indirect effect via proactive strategies [β = 0.18, *p* = 0.006, 95th C.I. (0.420–1.22)]. The latent endogenous variable compassion strategies reported a statistically significant total and positive standardized total effect on work engagement [β = 0.37, *p* = 0.014, 95th C.I. (0.701–1.28)], composed of both a significant direct effect on work engagement [β = 0.15, *p* = 0.013, 95th C.I. (2.26–4.47)] and a significant indirect effect via proactive strategies [β = 0.22, *p* = 0.009, 95th C.I. (0.148–0.810)]. Compassion strategies also reported a statistically significant direct effect on proactive strategies: β = 0.39, *p* = 0.011, 95th C.I. (0.047–0.192). With regard to the proactive strategies, the results evidenced their medium, positive, and statistically significant total effect on work engagement [β = 0.56, *p* = 0.013, 95th C.I. (2.26–4.47)], meaning that the greater teachers use self- and co-regulated strategies, the higher their levels of work engagement.

## Discussion

In the present study, we set out to investigate the network of relationships between subjective happiness, compassion, work engagement, and the use of proactive strategies (both self- and co-regulated) in a sample of early childhood teachers. We found that subjective happiness and compassion had direct positive effects on work engagement. Furthermore, the effect of subjective happiness and compassion on work engagement was mediated by the deployment of proactive strategies. Early childhood teachers’ self- and co-regulation (i.e., their ability to identify and use resources for coping with stressors as well as to seek and receive social support from colleagues) also had a direct positive effect on work engagement, lending support to the idea that using such strategies enhances early childhood teachers’ degree of involvement in their work. We now discuss the different roles played by the variables under study considering their direct and indirect effects on levels of work engagement.

### The Direct and Indirect Effects of Subjective Happiness and Compassion on Work Engagement

Our data confirm previous findings reported in the literature about, on the one hand, the relationship between positive emotions (triggered by subjective happiness and compassion at work) and proactive strategies and, on the other, the link between the use of proactive strategies and personal involvement in one’s work. For example, the present results are in line with studies reporting the beneficial effects of positive emotions on individuals’ behavioral and cognitive repertoires (Fredrickson, 2001), as well as the effects of personal and relational proactive strategies on engagement (Salmela-Aro et al., 2011).

Considering that work engagement is the opposite of burnout, the present findings are also in line with previous research identifying subjective happiness and compassion as key personal resources for coping with work-related stress and burnout (Field and Buitendach, 2011; Huynh et al., 2014; Bakker and Oerlemans, 2016). Vice versa, past studies found that depressive symptoms are negatively related to work engagement (Upadyaya et al., 2016), again indirectly confirming the importance of subjective happiness as a contributor to work engagement. Indeed, subjective happiness has been shown to enhance both teaching and broader life experience, generating work engagement by fostering more effective deployment of job resources (Bakker et al., 2014). Nevertheless, to the best of our knowledge, subjective happiness has not previously been investigated as a potential antecedent of work engagement among kindergarten teachers.

A further key finding of this study was that compassion had a positive effect on work engagement. Again, this finding is in line with previous studies showing compassion to be a correlate of work commitment (Mason et al., 2014; Eldor and Shoshani, 2016). In this respect, Tremblay and Messervey (2011) argued that compassion may generate work engagement by buffering the impact of job demands on work-related strain. More generally, personal resources such as subjective happiness and compassion may determine how job resources—the strongest predictors of work engagement—are perceived (Lorente et al., 2014; Diener et al., 2015).

The effect of compassion and subjective happiness on work engagement is greatly increased when mediated by coping strategies.

Overall, our findings suggest that the self-reported deployment of self- and co-regulated proactive strategies on the part of early childhood teachers is positively associated with their work-related well-being, in terms of self-perceived work engagement. Existing studies suggest that teachers who experience positive involvement in their work are more inclined to draw on active and reflective problem-solving strategies (Soini et al., 2010; Simbula et al., 2011). Furthermore, experiencing positive emotions prompts more creative and flexible use of available coping strategies (Fredrickson, 2001), enabling teachers to tackle challenges in innovative and original ways (Lyubomirsky et al., 2005; Linnenbrink-Garcia et al., 2011).

In keeping with the present findings, adopting proactive strategies has been associated with more effective regulation of behavior and greater adaptability to the work environment on the part of teachers. In fact, the deployment of proactive strategies is related to the ability to manage work-related burden and feel positively involved in it (Butler, 2007; Salmela-Aro et al., 2011; Devos et al., 2012), even when workload is perceived as particularly intense (Pomaki et al., 2010).

The literature suggests that the use of proactive strategies may also be associated with lower levels of perceived stress and more effective use of existing personal and relational resources on the part of teachers (De Caroli and Sagone, 2012; Pietarinen et al., 2013; Bermejo-Toro et al., 2016; Tikkanen et al., 2017). A proactive personal attitude is linked with work engagement and takes the form—for example—of actively seeking support from one’s teaching colleagues and setting stimulating goals (Bakker et al., 2012). Pietarinen et al. (2013) examined the relationship—in a sample of teachers—between their deployment of proactive strategies, workplace difficulties, and perceived adaptability.

The present findings confirm the role of proactive strategies as protective factors. Again, this bears out previous research suggesting that when teachers are able to use multiple coping strategies, this protects the teacher community from emotional exhaustion (Kyriacou, 2001; Howard and Johnson, 2004; Austin et al., 2005; Carmona et al., 2006).

Thus, self-perceived work engagement may be seen as a social outcome that is dependent on teachers’ subjective well-being at work and may be significantly enhanced by the deployment of self- and co-regulative strategies on the part of the community of education practitioners. Using proactive strategies allows teachers to simultaneously regulate their own behavior and their working environment, thus enhancing working environment fit.

*Ad hoc* interventions for fostering the use of proactive strategies should be designed, with a view to reducing teachers’ risk of burnout and increasing their engagement and positive involvement in the educational setting (Pietarinen et al., 2013).

## Conclusion and Implications

The present study advances our understanding of early childhood teachers’ work engagement, by evaluating the contributions of subjective happiness, compassion, and proactive strategies to work engagement within a single model. It shows that subjective happiness and compassion at work trigger positive feelings, which in turn contribute to enhancing pre-school teachers’ attitudes and work outcomes. Another key outcome is that the dispositional variables happiness and compassion need to be mediated by proactive strategies, undertaken both individually and jointly with colleagues, for teachers to attain a fuller sense of work engagement. A novel aspect of the current research is that it specifically analyzed the work-related well-being of a group of early childhood teachers, a population whose work engagement has been little investigated in the literature to date (Hall-Kenyon et al., 2014; Cumming, 2017). Nonetheless, it is well known that children’s well-being is closely related to teachers’ well-being and work engagement: for example, when teachers perceive their work community in a positive light, this is associated with better classroom teaching quality (McGinty et al., 2008). Early education environments are characterized by multiple stressors including the children’s needs, relationships with families and colleagues, and organizational issues (Kelly and Berthelsen, 1995; Rentzou, 2012; Jovanovic, 2013; Faulkner et al., 2014; Nislin et al., 2016), with potentially detrimental repercussions on teacher–child interaction and infant development (e.g., De Schipper et al., 2008).

In conclusion, the current findings suggest that early childhood teachers are more inclined to reinforce their work engagement by drawing on positive workplace relationships than by relying on their dispositional characteristics. Positive interpersonal relationships in the school setting promote work engagement and protect early childhood teachers from the risk of burnout. Based on these findings, we strongly recommend intervention in the domains of personal resources and work-related well-being in the interest of optimizing early childhood teachers’ work engagement.

## Data Availability Statement

All datasets generated for this study are included in the manuscript/supplementary files.

## Ethics Statement

The study involving human participants was reviewed and approved by the Scientific Board of LUMSA University, Rome. The participants provided their written informed consent to participate in this study.

## Author Contributions

SD, CF, and PB designed and carried out the study, contributed to the analysis of the results and to the writing of the manuscript. BR, FB, AP, and JM collected the data, and contributed to the analysis of the results and to the writing of the manuscript. PB, SD, and JM supervised the study design and the manuscript draft.

## Conflict of Interest

The authors declare that the research was conducted in the absence of any commercial or financial relationships that could be construed as a potential conflict of interest.
